# The Relation of Modern Pathology and Pathological Anatomy to Practical Medicine

**Published:** 1890-11-15

**Authors:** 


### The Relation of Modern Pathology and Pathological Anatomy to Practical Medicine.

In the development of medicine which has occurred within the last fifty years, the study of the changes from normal structure and relations which occur in different parts and organs of the body holds a conspicuous place.

Modern pathology, in its broad sense, as studied and taught in some of the laboratories or institutions specially devoted to that subject, has become almost a science in itself, or, rather, it has taken position as one of the branches of general biological study; and many of the investigations carried on by its votaries, as well as much of its recent literature, have no very evident relations to practical medicine. Modern pathologists are studying the processes of disease, and not merely the results; in other words, they are investigating variations in the intensity or mode of action of normal, or physiological, processes as these affect, or are affected by, minute histological elements or their aggregation into tissues or organs. Some of the results thus obtained have already been of much importance in improving means of diagnosis, in enabling the physician to foretell results, and, to a more limited extent, in practical therapeutics, more especially in the surgical field; other results, though not yet of practical application, give promise of being so in the near future; but the modern scientific pathologist does the greater part of his work without any special reference to its probable or possible influence on methods of treating disease. This is as it should be, nothing could be more foolish than to attempt to limit the investigations of the pathologist to questions which seem to have some practical bearing on therapeutics, because no man can say what bearings the result of any increase in knowledge of biological processes, whether normal or abnormal, may have upon the future daily routine work of the practioner or nurse, but it does not follow that every student or practitioner of medicine should be expected to be familiar with the methods or results of pathological laboratory work, and much less that he should try to fit himself to make investigations of this kind. The average graduate of last year would require from two to four years' special instruction and study before he could be considered as being a good pathologist, even if he had special inclination to that branch of science, and much of the modern literature of the subject would be almost unintelligible to the great majority of practitioners of twenty years' standing. Very few practitioners could make a post-mortem and describe accurately the lesions found in cases involving localised degeneration of the central nervous system—is it sufficiently desirable that they should be able to do this to warrant their giving the time and labour required to obtain the necessary information?

Considered as a matter affecting their practice, the answer must be no, because the time and opportunities afforded them to master the details of their art are limited, and as a rule, insufficient, and should be used mainly, or exclusively for acquiring the knowledge and technique which will be most frequently demanded of them by their patients. There is a vast amount of such knowledge and technique pertaining to the different specialties which it has not been worth the while of the average medical student to attempt to acquire, and which will still remain so although the term of study has been extended to five years. The additional time required by the new regulation is all needed for clinical study by at least 75 per cent. of our medical graduates. By clinical study we do not mean the listening to clinical lectures, but the personal investigation and treatment of cases under skilled direction, the acquiring of the sense of responsibility without the burden of doing it alone and unaided.

It will be seen that we consider modern pathology as a specialty, and a highly developed specialty, of which only the broad general principles should be acquired by the student, and which should be left to be dealt with according to the leisure, opportunities, and taste of each physician after graduation.

By all means let us have pathological laboratories and skilled pathologists, who are not only familiar with what is going on in this line of work all over the world, but who are making original investigations themselves. But do not let us insist that every physician must be skilled in the technique of physiological or pathological histology, or shall be able at sight to give the correct generic name of any monstrosity or abnormity that may be presented to him.

It is true that he might have a case the first week after he opens his office, in which such knowledge would be useful to him; he might be called on to make a post-mortem in a case of judicial inquiry, which would involve the giving of an opinion as to the nature of certain microscopical appearances in the brain and spinal cord, and that if he could answer correctly and convincingly it might be the first step to fame and fortune; but the probabilities are immensely against it. It is an engineering maxim that it is not worth while to build a bridge in such a manner as to ensure that it will resist a tornado, and so it is not worth while to try to educate a physician so that he shall be prepared to meet every possible emergency, and still less worth while to attempt to educate all physicians to this degree of perfection. This is by no means to be understood that it is not worth while for practitioners to study modern pathology. On the contrary, every practitioner should strive to thoroughly master what is known with regard to the pathology of at least one organ or system, and this without special reference to its practical bearings, but for the sake of mental exercise and as a matter of pleasure. To this, as to other branches of science connected with medicine, the words of Sir James Paget apply: "He that ceases to gain knowledge is always losing it, and he that is not a student whilst he is in practice is sure to become less fitted for his work every year he lives."

---

Turning Over New Leaves.—The authoress of "The Modern Malady," whose good work in many directions and on many occasions we fully appreciate, sends us a letter in reference to a note which appeared in The Hospital on the 1st inst. (p. 77) under the above heading. The letter is dated Valentines, Ilford, and is as follows:—"In the current number of The Hospital you refer me to the first 100 pages of 'The Modern Malady' for proof of your reviewer's assertion that one of the objects aimed at by me is 'To show up massage and allied treatments as ridiculous for the relief of neurasthenia.' I have referred to them accordingly, but I am unable to find, from the first page to the last, one word written against well-performed, painless massage as a remedy for neurasthenia. On page 72, however, I find both massage and electricity spoken of as valuable remedies, and I find a preface by the founder and director of the School of Massage and Electricity, in which those modes of treatment are strongly recommended. Two or three instances are, indeed, given of the abuse of massage and allied treatments, and as such cases cause useful remedies to be brought into disrepute, attention cannot be too urgently directed to them. —I am, etc., Cyril Bennett."

